# Prevalence of Retrocaecal Appendix among Patients with Appendicitis in a Tertiary Care Hospital of Nepal

**DOI:** 10.31729/jnma.4370

**Published:** 2019-06-30

**Authors:** Sanzida Khatun, Dipendra Thakur, Diwakar Kumar Shah

**Affiliations:** 1 Department of Anatomy, Nobel Medical College, Biratnagar, Nepal; 2 Department of General Surgery, Nobel Medical College, Biratnagar, Nepal

**Keywords:** *appendicitis*, *appendix*, *positions of appendix*, *vermiform appendix*

## Abstract

**Introduction:**

Appendicitis is one of the most common causes of acute abdomen. The clinical presentation of appendicitis and its susceptibility to acute inflammation may be affected by the length and position of vermiform appendix. Length and position of appendix are variable. The aim of the study was to find the prevalence of retrocaecal appendix among patients undergoing appendectomy for appendicitis.

**Methods:**

A descriptive cross-sectional study was performed in 264 patients undergoing appendectomy in Department of Surgery, Nobel Medical College, Biratnagar, Nepal from 1st May, 2018 to 15th May, 2019. Ethical approval was taken. Simple random sampling was done. The position of appendix was noted before appendectomy. Subgroup analysis was done on the basis of gender and length of appendix recorded in centimeters with a measuring scale immediately after removal of appendix. Data was collected in excel and was analyzed in SPSS version 16.

**Results:**

Prevalence of retrocaecal appendix among patients with appendicitis was 95 (35.98%). Similarly, other positions noted were pelvic in 67 (25.37%), post-ileal in 61 (23.10%), pre-ileal in 11 (4.16%) and subcaecal in 30 (11.36%) individuals. The length of appendix ranged from 1.7 cm to 14.7 cm. The mean length was 8.67±2.44 cm.

**Conclusions:**

The most common position of appendix in patients with appendicitis is retrocaecal position followed by pelvic position in both males and females.

## INTRODUCTION

The vermiform appendix, a long, narrow tube like structure, arises 2cm below the ileocaecal opening from the posteromedial wall of caecum.^1^ Its length and position are variable. The length ranges from 1 to 25 cm.^2^ The five most common positions of appendix include pelvic, retrocaecal, subcaecal, pre-ileal and post-ileal positions.^1^

Appendicitis is one of the most common causes of acute abdomen.^3^ The clinical presentation of appendicitis and its susceptibility to acute inflammation may be affected by the length and position of appendix.^4,5^ However, some studies show that position does not alter the clinical presentation.^6,7^ Although few studies have been done in appendicitis in Nepalese population, the length and position of inflamed appendix have not been reported in Nepal.

The aim of the study was to find the prevalence of retrocecal appendix among patients with appendicitis undergoing appendectomy in a tertiary care hospital of Nepal.

## METHODS

A descriptive cross-sectional study was performed in patients undergoing appendectomy in Department of Surgery, Nobel Medical College, Biratnagar, Nepal. The study was conducted from 1^st^ May, 2018 to 15^th^ May, 2019.

The ethical approval was received from Institutional Review Committee of Nobel Medical College. Individuals who had undergone abdominal surgery previously were excluded from the study. A written informed consent was obtained from the subjects or the guardians of the pediatric patients.

Sample size was calculated using the following formula,


$$\text{n}={\text{Z}}^{2}\times (\text{p}\times \text{q})/{\text{d}}^{2}$$

where,

n = sample size
p = prevalence, 45% (educated guess)
q = 1-p
d = margin of error, 6%
Z = 1.96 at 95% CI

Based on the above formula, the minimum sample size at 95% confidence interval with 6% error was calculated to be 264 and thus study was conducted in 264 patients. Simple random sampling was done.

The position of appendix was noted before appendectomy and the length of appendix was recorded in centimeters (cm) with a measuring scale immediately after removal of appendix. The data were collected and analyzed using Excel and Statistical Package for Social Sciences 16 software respectively. Subgroup analysis was done on the basis of age, gender and length of appendix.

## RESULTS

Prevalence of retrocaecal appendix was 95 (35.98%). Similarly, other positions noted were pelvic in 67 (25.37%) patients, post-ileal in 61 (23.10%) patients, pre-ileal in 11 (4.16%) patients and subcaecal in 30 (11.36%) patients. The most common position of appendix was retrocaecal position followed by pelvic, post-ileal, subcaecal and pre-ileal positions (Table 1).

**Table 1.** Frequency of different positions of appendix in both sexes.

Position of Appendix	Male n (%)	Female n (%)	Total n (%)
Pelvic	43 (25.6%)	24 (25.0%)	67 (25.4%)
Retrocaecal	61 (36.3%)	34 (35.4%)	95 (36.0%)
Subcaecal	20 (11.9%)	10 (10.4%)	30 (11.4%)
Pre-ileal	6 (3.6%)	5 (5.2%)	11 (4.2%)
Post-ileal	38 (22.6%)	23 (24.0%)	61 (23.1%)
Total	168 (100.0%)	96 (100.0%)	264 (100.0%)

**Figure 1.**
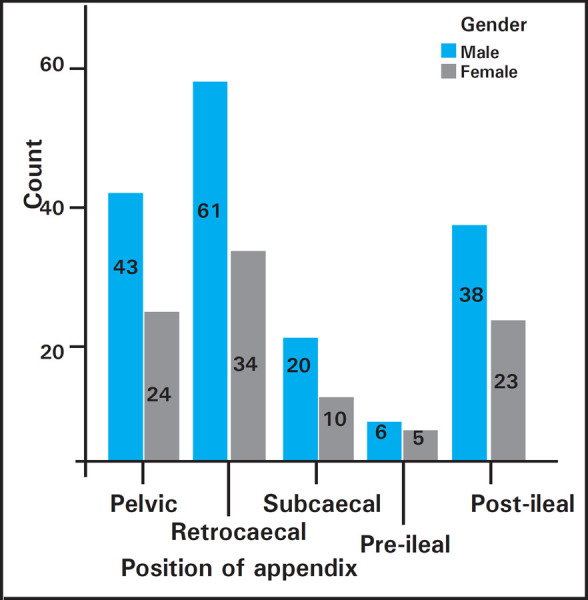
Bar Diagram showing distribution of positions of appendix in both sexes.

The study was performed in 264 patients where 168 (63.6%) were males and 96 (36.4%) were females. The mean age of the study population was 28.6 years. The length of appendix ranged from 1.7 cm to 14.7 cm. The mean length was 8.67 ± 2.44 cm. The mean length of appendix was 8.74± 2.41 cm in males and 8.55 ± 2.48 cm in females. The most common position was retrocaecal in both sexes (Figure 1).

## DISCUSSION

The length and position of the vermiform appendix are inconsistent in humans. It is an organ which is mobile and can change its position in various situations.^8^ This study has demonstrated that most commonly found position of appendix in subjects with appendicitis is retrocaecal followed by pelvic then, post-ileal, paracolic and least prevalent is pre-ileal. In consistence to our finding, the findings of Clegg-Lamptey,^9^ Pau1,^10^ Wakeley,^11^ Mwachaka^12^ and Setty^13^ reveal that retrocaecal is the most common position of appendix followed by pelvic position.

In contrast to the finding of this study, the findings of Ghorbani,^14^ Rahman,^15^ Ahmed,^16^ Tofighil^7^ and Golalipourl° show that the most common position of appendix is pelvic position. The pelvic position of appendix favors pressure free surrounding and in retrocaecal position, the appendix is kinked by loaded caecum or ascending colon.^19^ Ghorbani illustrates in his study that 75% of appendix are anterior to caecum which favors early diagnosis and short hospitalization.^14^

Searle described in his study that appendix achieves adult proportion after 3 years of age and does not continue to grow throughout childhood.^2^° The current study estimated 8.67 ± 2.44 cm mean length of the appendix. There is significant association between length of appendix and different age groups. The mean length of appendix is 8.74 ± 2.41 cm in males and 8.55±2.48 cm in females. The mean lengths of appendix in male and female was 9.12 cm and 8.03 cm, respectively in a study performed in Iran.^14^ In a cadaveric study in Kenya, the mean length of appendix estimated was 7.65 ± 2.36 cm.^12^ The mean length of appendix was lower, 6.52 cm in males and 6.28 cm in females, in a study performed in India. In the same study, the mean length in fetus was found to be 2.0 to 5.0 cm.^13^ In a post mortem study done in 4,680 subjects, it was found that the length of 61 % of appendices ranged from 6.00-9.00 cm.^21^ An appendix, 28cm long, in retrocaecal position was reported in adult female cadaver in India.^22^

It has been hypothesized from previous studies that appendix lying anterior to caecum or in pelvic position prevents kinking and favors early diagnosis of appendicitis, whereas, retrocaecal position may produce kinking thus compromising the blood supply to appendix.^14,19^ A larger sample of appendicitis subjects will help establish whether retrocaecal or pelvic positions support or prevent appendicitis. Further researches are yet to be done to understand the relation between particular position of appendix and occurrence of appendicitis.

## CONCLUSIONS

The most common position of appendix in patients with appendicitis is retrocaecal position followed by pelvic position in both males and females.
